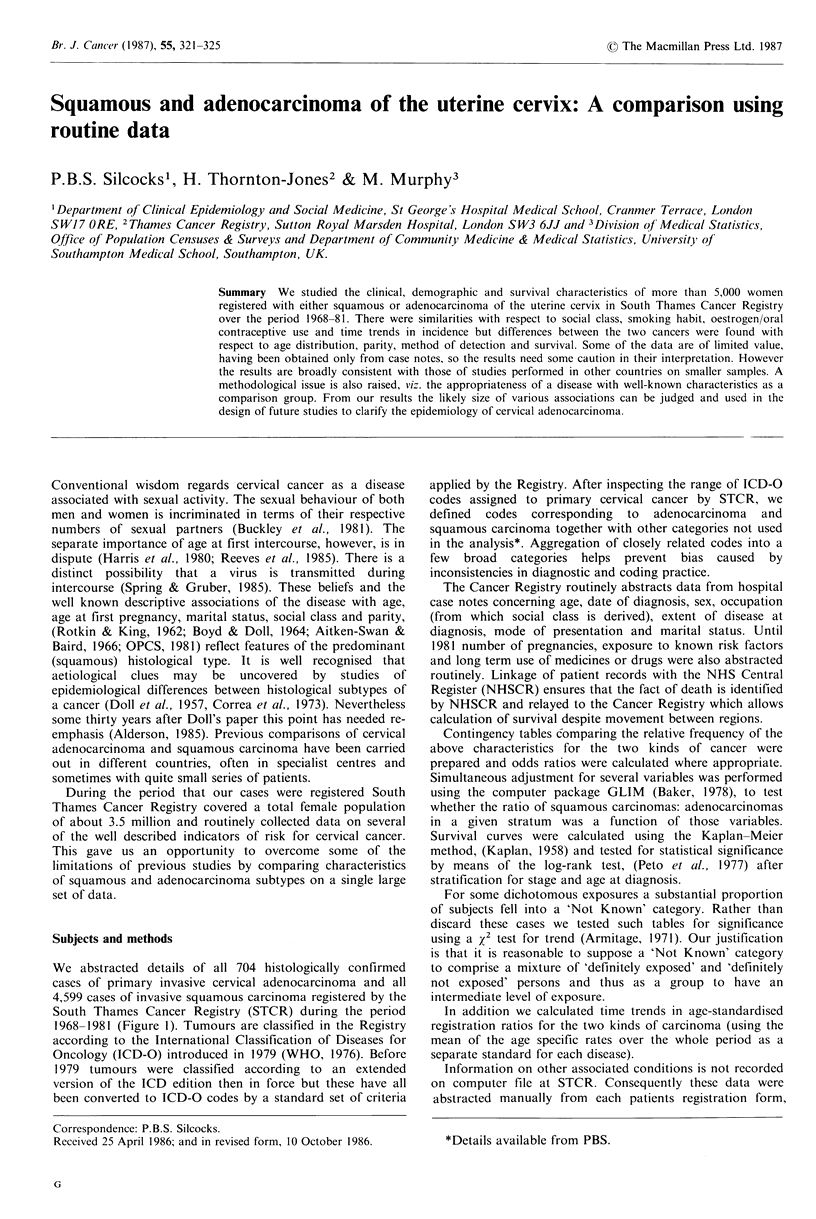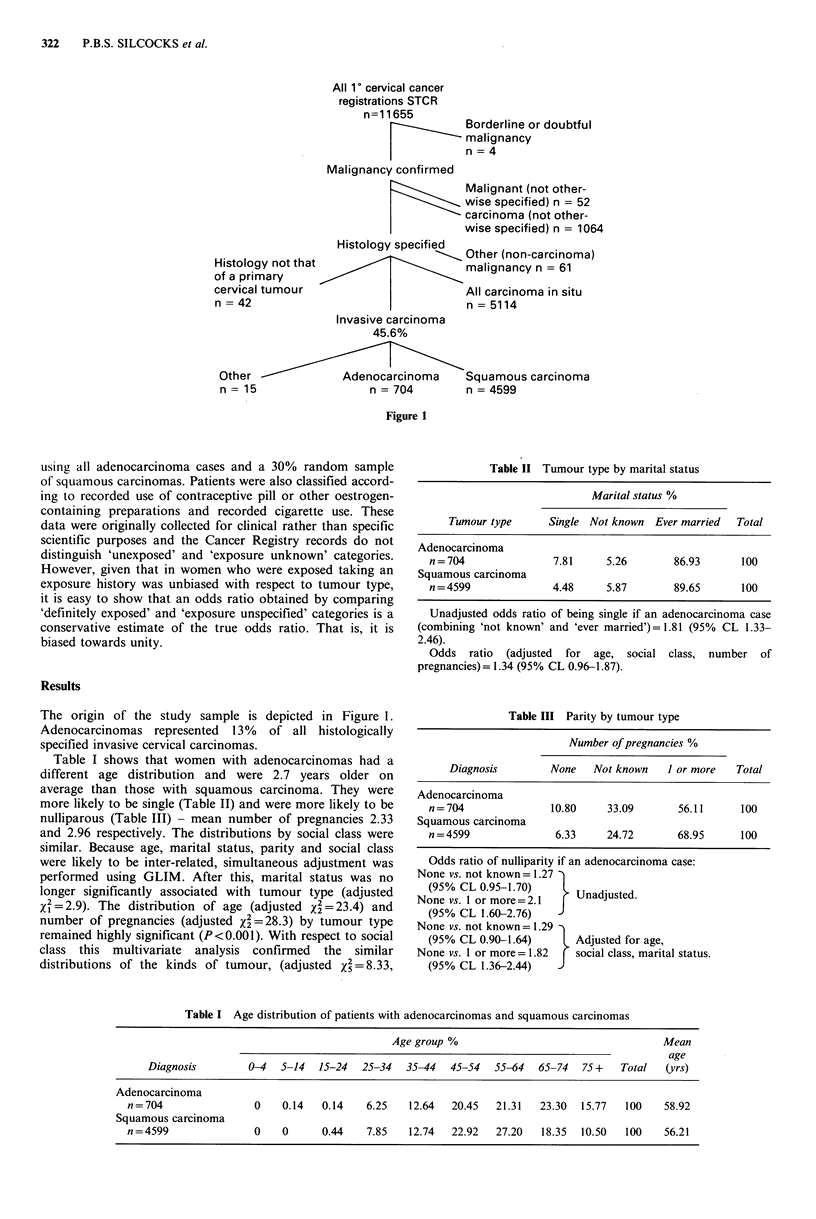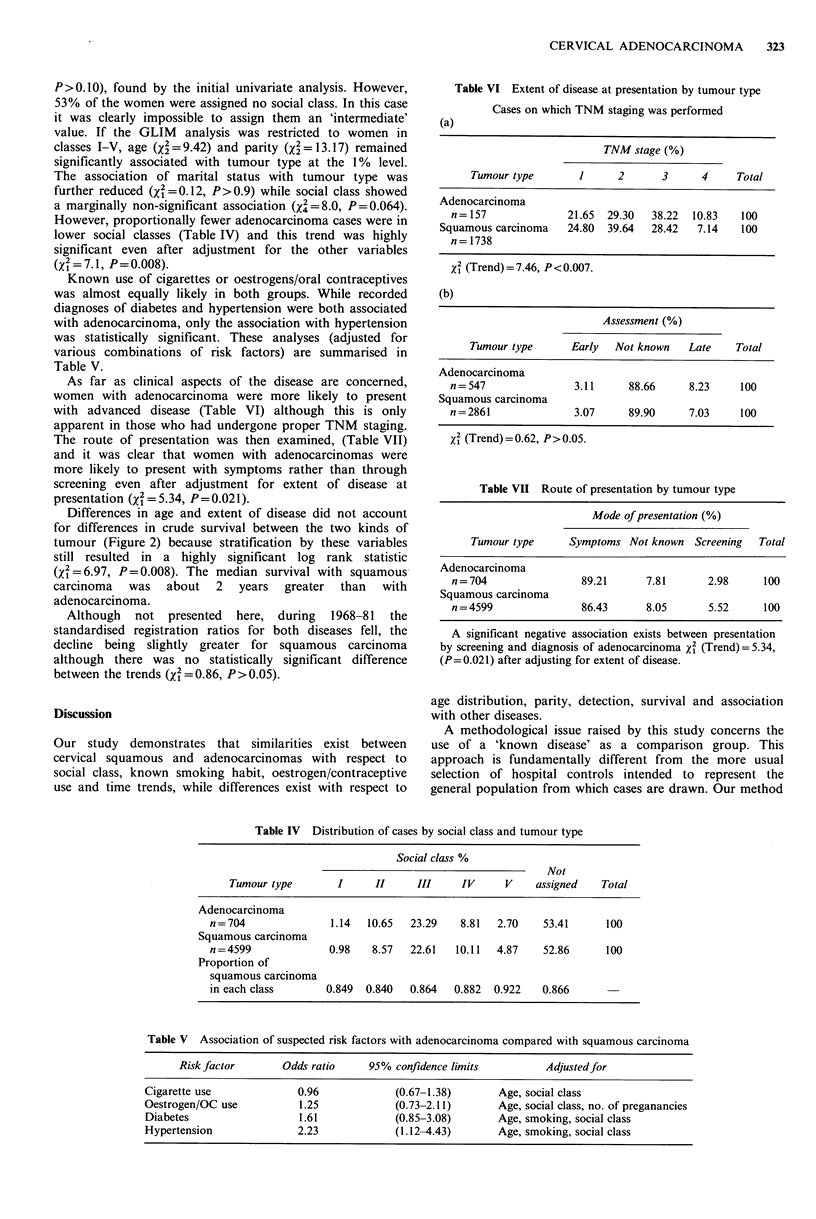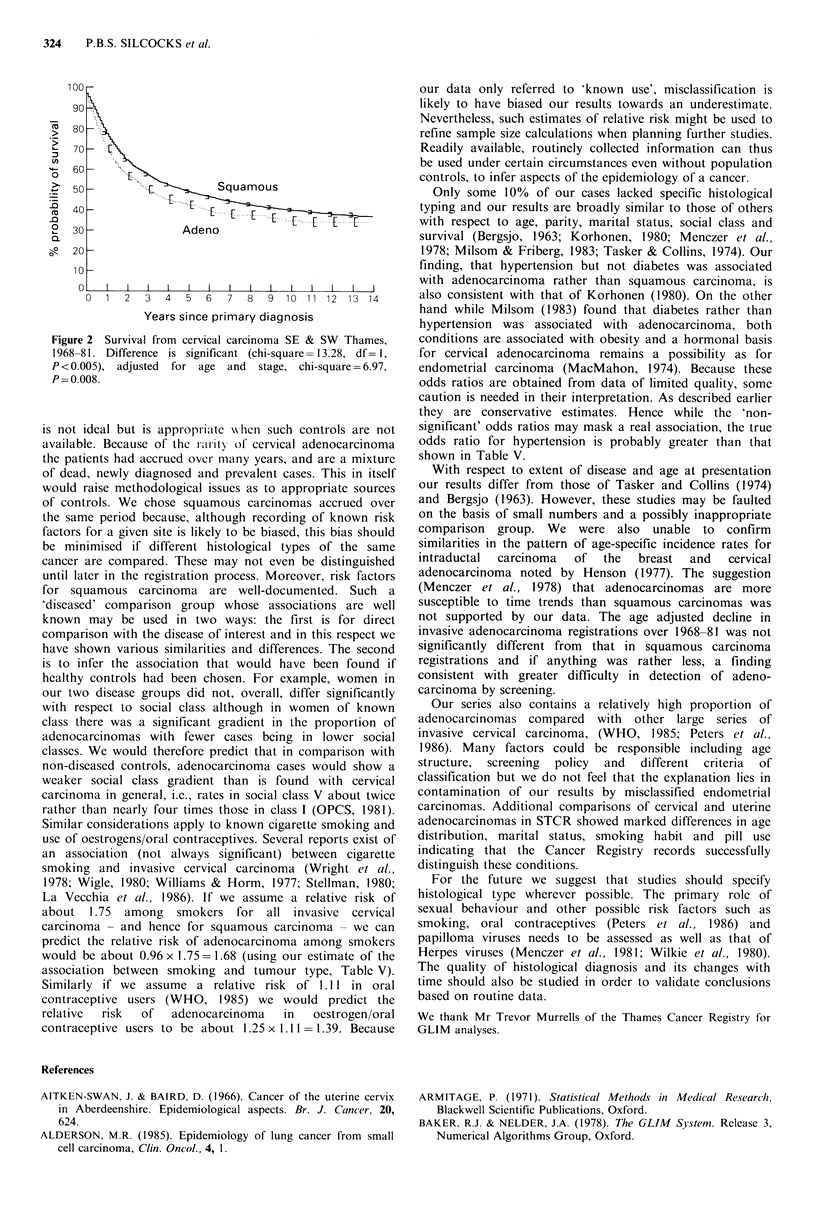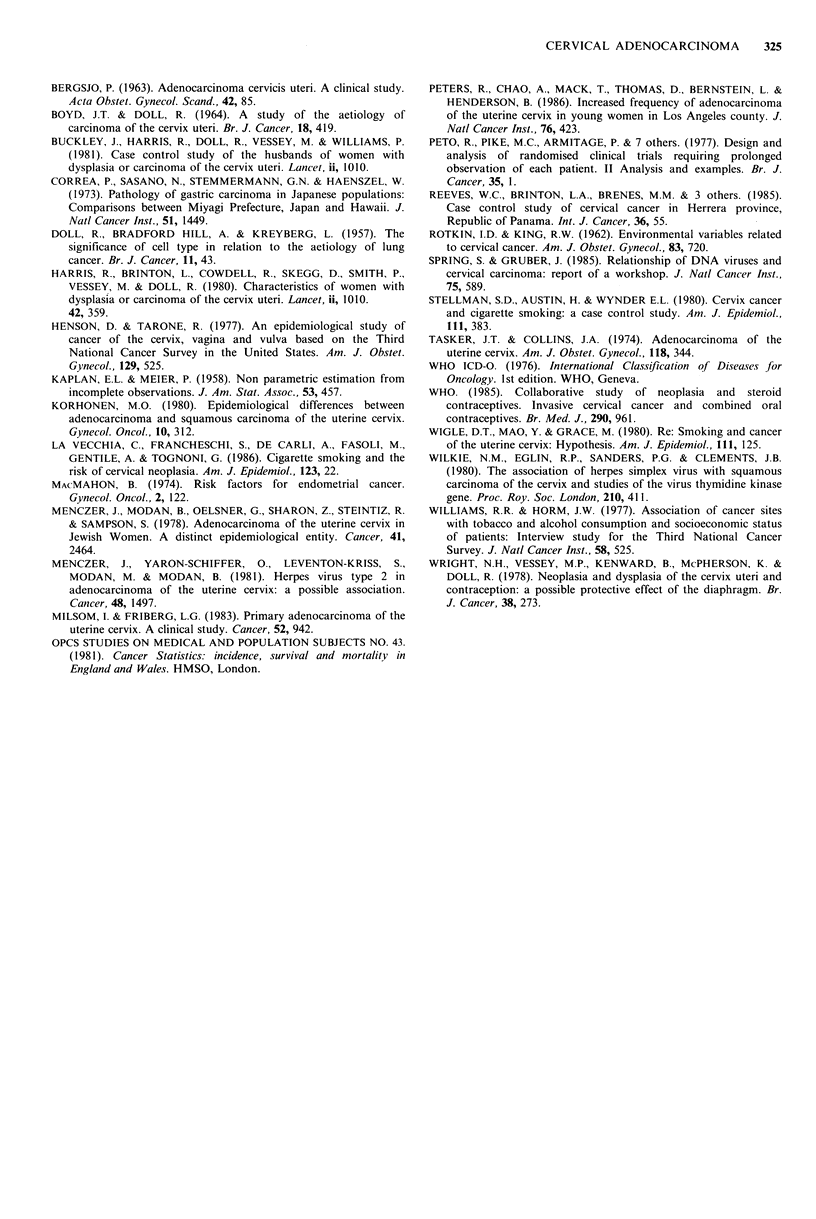# Squamous and adenocarcinoma of the uterine cervix: a comparison using routine data.

**DOI:** 10.1038/bjc.1987.63

**Published:** 1987-03

**Authors:** P. B. Silcocks, H. Thornton-Jones, M. Murphy

## Abstract

We studied the clinical, demographic and survival characteristics of more than 5,000 women registered with either squamous or adenocarcinoma of the uterine cervix in South Thames Cancer Registry over the period 1968-81. There were similarities with respect to social class, smoking habit, oestrogen/oral contraceptive use and time trends in incidence but differences between the two cancers were found with respect to age distribution, parity, method of detection and survival. Some of the data are of limited value, having been obtained only from case notes, so the results need some caution in their interpretation. However the results are broadly consistent with those of studies performed in other countries on smaller samples. A methodological issue is also raised, viz. the appropriateness of a disease with well-known characteristics as a comparison group. From our results the likely size of various associations can be judged and used in the design of future studies to clarify the epidemiology of cervical adenocarcinoma.


 © The Macmillan Press Ltd. 1987

### Squamous and adenocarcinoma of the uterine cervix: A comparison using routine data

P.B.S. Silcocks[1], H. Thornton-Jones[2] & M. Murphy[3]

[1]*Department of Clinical Epidemiology and Social Medicine, St George's Hospital Medical School, Cranmer Terrace, London SW17 0RE,* [2]*Thames Cancer Registry, Sutton Royal Marsden Hospital, London SW3 6JJ and* [3]*Division of Medical Statistics, Office of Population Censuses & Surveys and Department of Community Medicine & Medical Statistics, University of Southampton Medical School, Southampton, UK.*

**Summary** We studied the clinical, demographic and survival characteristics of more than 5,000 women registered with either squamous or adenocarcinoma of the uterine cervix in South Thames Cancer Registry over the period 1968–81. There were similarities with respect to social class, smoking habit, oestrogen/oral contraceptive use and time trends in incidence but differences between the two cancers were found with respect to age distribution, parity, method of detection and survival. Some of the data are of limited value, having been obtained only from case notes, so the results need some caution in their interpretation. However the results are broadly consistent with those of studies performed in other countries on smaller samples. A methodological issue is also raised, *viz.* the appropriateness of a disease with well-known characteristics as a comparison group. From our results the likely size of various associations can be judged and used in the design of future studies to clarify the epidemiology of cervical adenocarcinoma.

Conventional wisdom regards cervical cancer as a disease associated with sexual activity. The sexual behaviour of both men and women is incriminated in terms of their respective numbers of sexual partners (Buckley *et al.*, 1981). The separate importance of age at first intercourse, however, is in dispute (Harris *et al.*, 1980; Reeves *et al.*, 1985). There is a distinct possibility that a virus is transmitted during intercourse (Spring & Gruber, 1985). These beliefs and the well known descriptive associations of the disease with age, age at first pregnancy, marital status, social class and parity, (Rotkin & King, 1962; Boyd & Doll, 1964; Aitken-Swan & Baird, 1966; OPCS, 1981) reflect features of the predominant (squamous) histological type. It is well recognised that aetiological clues may be uncovered by studies of epidemiological differences between histological subtypes of a cancer (Doll *et al.*, 1957, Correa *et al.*, 1973). Nevertheless some thirty years after Doll's paper this point has needed re-emphasis (Alderson, 1985). Previous comparisons of cervical adenocarcinoma and squamous carcinoma have been carried out in different countries, often in specialist centres and sometimes with quite small series of patients.

During the period that our cases were registered South Thames Cancer Registry covered a total female population of about 3.5 million and routinely collected data on several of the well described indicators of risk for cervical cancer. This gave us an opportunity to overcome some of the limitations of previous studies by comparing characteristics of squamous and adenocarcinoma subtypes on a single large set of data.

### Subjects and methods

We abstracted details of all 704 histologically confirmed cases of primary invasive cervical adenocarcinoma and all 4,599 cases of invasive squamous carcinoma registered by the South Thames Cancer Registry (STCR) during the period 1968–1981 (Figure 1). Tumours are classified in the Registry according to the International Classification of Diseases for Oncology (ICD-O) introduced in 1979 (WHO, 1976). Before 1979 tumours were classified according to an extended version of the ICD edition then in force but these have all been converted to ICD-O codes by a standard set of criteria applied by the Registry. After inspecting the range of ICD-O codes assigned to primary cervical cancer by STCR, we defined codes corresponding to adenocarcinoma and squamous carcinoma together with other categories not used in the analysis*. Aggregation of closely related codes into a few broad categories helps prevent bias caused by inconsistencies in diagnostic and coding practice.

The Cancer Registry routinely abstracts data from hospital case notes concerning age, date of diagnosis, sex, occupation (from which social class is derived), extent of disease at diagnosis, mode of presentation and marital status. Until 1981 number of pregnancies, exposure to known risk factors and long term use of medicines or drugs were also abstracted routinely. Linkage of patient records with the NHS Central Register (NHSCR) ensures that the fact of death is identified by NHSCR and relayed to the Cancer Registry which allows calculation of survival despite movement between regions.

Contingency tables comparing the relative frequency of the above characteristics for the two kinds of cancer were prepared and odds ratios were calculated where appropriate. Simultaneous adjustment for several variables was performed using the computer package GLIM (Baker, 1978), to test whether the ratio of squamous carcinomas: adenocarcinomas in a given stratum was a function of those variables. Survival curves were calculated using the Kaplan–Meier method, (Kaplan, 1958) and tested for statistical significance by means of the log-rank test, (Peto *et al.*, 1977) after stratification for stage and age at diagnosis.

For some dichotomous exposures a substantial proportion of subjects fell into a 'Not Known' category. Rather than discard these cases we tested such tables for significance using a $\chi^2$ test for trend (Armitage, 1971). Our justification is that it is reasonable to suppose a 'Not Known' category to comprise a mixture of 'definitely exposed' and 'definitely not exposed' persons and thus as a group to have an intermediate level of exposure.

In addition we calculated time trends in age-standardised registration ratios for the two kinds of carcinoma (using the mean of the age specific rates over the whole period as a separate standard for each disease).

Information on other associated conditions is not recorded on computer file at STCR. Consequently these data were abstracted manually from each patients registration form,

Correspondence: P.B.S. Silcocks.
Received 25 April 1986; and in revised form, 10 October 1986.

*Details available from PBS.

**Figure 1**

using all adenocarcinoma cases and a 30% random sample of squamous carcinomas. Patients were also classified according to recorded use of contraceptive pill or other oestrogen-containing preparations and recorded cigarette use. These data were originally collected for clinical rather than specific scientific purposes and the Cancer Registry records do not distinguish 'unexposed' and 'exposure unknown' categories. However, given that in women who were exposed taking an exposure history was unbiased with respect to tumour type, it is easy to show that an odds ratio obtained by comparing 'definitely exposed' and 'exposure unspecified' categories is a conservative estimate of the true odds ratio. That is, it is biased towards unity.

### Results

The origin of the study sample is depicted in Figure 1. Adenocarcinomas represented 13% of all histologically specified invasive cervical carcinomas.

Table I shows that women with adenocarcinomas had a different age distribution and were 2.7 years older on average than those with squamous carcinoma. They were more likely to be single (Table II) and were more likely to be nulliparous (Table III) – mean number of pregnancies 2.33 and 2.96 respectively. The distributions by social class were similar. Because age, marital status, parity and social class were likely to be inter-related, simultaneous adjustment was performed using GLIM. After this, marital status was no longer significantly associated with tumour type (adjusted $\chi_1^2=2.9$). The distribution of age (adjusted $\chi_2^2=23.4$) and number of pregnancies (adjusted $\chi_2^2=28.3$) by tumour type remained highly significant ($P<0.001$). With respect to social class this multivariate analysis confirmed the similar distributions of the kinds of tumour, (adjusted $\chi_5^2=8.33$,

**Table II** Tumour type by marital status

| Tumour type | Marital status % Single | Not known | Ever married | Total |
|---|---|---|---|---|
| Adenocarcinoma *n*=704 | 7.81 | 5.26 | 86.93 | 100 |
| Squamous carcinoma *n*=4599 | 4.48 | 5.87 | 89.65 | 100 |

Unadjusted odds ratio of being single if an adenocarcinoma case (combining 'not known' and 'ever married') = 1.81 (95% CL 1.33–2.46).

Odds ratio (adjusted for age, social class, number of pregnancies) = 1.34 (95% CL 0.96–1.87).

**Table III** Parity by tumour type

| Diagnosis | Number of pregnancies % None | Not known | 1 or more | Total |
|---|---|---|---|---|
| Adenocarcinoma *n*=704 | 10.80 | 33.09 | 56.11 | 100 |
| Squamous carcinoma *n*=4599 | 6.33 | 24.72 | 68.95 | 100 |

Odds ratio of nulliparity if an adenocarcinoma case:

None *vs.* not known = 1.27 (95% CL 0.95–1.70); None *vs.* 1 or more = 2.1 (95% CL 1.60–2.76) — Unadjusted.

None *vs.* not known = 1.29 (95% CL 0.90–1.64); None *vs.* 1 or more = 1.82 (95% CL 1.36–2.44) — Adjusted for age, social class, marital status.

**Table I** Age distribution of patients with adenocarcinomas and squamous carcinomas

| Diagnosis | Age group % 0–4 | 5–14 | 15–24 | 25–34 | 35–44 | 45–54 | 55–64 | 65–74 | 75+ | Total | Mean age (yrs) |
|---|---|---|---|---|---|---|---|---|---|---|---|
| Adenocarcinoma *n*=704 | 0 | 0.14 | 0.14 | 6.25 | 12.64 | 20.45 | 21.31 | 23.30 | 15.77 | 100 | 58.92 |
| Squamous carcinoma *n*=4599 | 0 | 0 | 0.44 | 7.85 | 12.74 | 22.92 | 27.20 | 18.35 | 10.50 | 100 | 56.21 |

$P>0.10$), found by the initial univariate analysis. However, 53% of the women were assigned no social class. In this case it was clearly impossible to assign them an 'intermediate' value. If the GLIM analysis was restricted to women in classes I–V, age ($\chi_2^2=9.42$) and parity ($\chi_2^2=13.17$) remained significantly associated with tumour type at the 1% level. The association of marital status with tumour type was further reduced ($\chi_1^2=0.12$, $P>0.9$) while social class showed a marginally non-significant association ($\chi_4^2=8.0$, $P=0.064$). However, proportionally fewer adenocarcinoma cases were in lower social classes (Table IV) and this trend was highly significant even after adjustment for the other variables ($\chi_1^2=7.1$, $P=0.008$).

Known use of cigarettes or oestrogens/oral contraceptives was almost equally likely in both groups. While recorded diagnoses of diabetes and hypertension were both associated with adenocarcinoma, only the association with hypertension was statistically significant. These analyses (adjusted for various combinations of risk factors) are summarised in Table V.

As far as clinical aspects of the disease are concerned, women with adenocarcinoma were more likely to present with advanced disease (Table VI) although this is only apparent in those who had undergone proper TNM staging. The route of presentation was then examined, (Table VII) and it was clear that women with adenocarcinomas were more likely to present with symptoms rather than through screening even after adjustment for extent of disease at presentation ($\chi_1^2=5.34$, $P=0.021$).

Differences in age and extent of disease did not account for differences in crude survival between the two kinds of tumour (Figure 2) because stratification by these variables still resulted in a highly significant log rank statistic ($\chi_1^2=6.97$, $P=0.008$). The median survival with squamous carcinoma was about 2 years greater than with adenocarcinoma.

Although not presented here, during 1968–81 the standardised registration ratios for both diseases fell, the decline being slightly greater for squamous carcinoma although there was no statistically significant difference between the trends ($\chi_1^2=0.86$, $P>0.05$).

**Table VI** Extent of disease at presentation by tumour type

(a) Cases on which TNM staging was performed

| *Tumour type* | *TNM stage* (%) *1* | *2* | *3* | *4* | *Total* |
|---|---|---|---|---|---|
| Adenocarcinoma $n=157$ | 21.65 | 29.30 | 38.22 | 10.83 | 100 |
| Squamous carcinoma $n=1738$ | 24.80 | 39.64 | 28.42 | 7.14 | 100 |

$\chi_1^2$ (Trend) = 7.46, $P<0.007$.

(b)

| *Tumour type* | *Assessment* (%) *Early* | *Not known* | *Late* | *Total* |
|---|---|---|---|---|
| Adenocarcinoma $n=547$ | 3.11 | 88.66 | 8.23 | 100 |
| Squamous carcinoma $n=2861$ | 3.07 | 89.90 | 7.03 | 100 |

$\chi_1^2$ (Trend) = 0.62, $P>0.05$.

**Table VII** Route of presentation by tumour type

| *Tumour type* | *Mode of presentation* (%) *Symptoms* | *Not known* | *Screening* | *Total* |
|---|---|---|---|---|
| Adenocarcinoma $n=704$ | 89.21 | 7.81 | 2.98 | 100 |
| Squamous carcinoma $n=4599$ | 86.43 | 8.05 | 5.52 | 100 |

A significant negative association exists between presentation by screening and diagnosis of adenocarcinoma $\chi_1^2$ (Trend) = 5.34, ($P=0.021$) after adjusting for extent of disease.

### Discussion

Our study demonstrates that similarities exist between cervical squamous and adenocarcinomas with respect to social class, known smoking habit, oestrogen/contraceptive use and time trends, while differences exist with respect to age distribution, parity, detection, survival and association with other diseases.

A methodological issue raised by this study concerns the use of a 'known disease' as a comparison group. This approach is fundamentally different from the more usual selection of hospital controls intended to represent the general population from which cases are drawn. Our method

**Table IV** Distribution of cases by social class and tumour type

| *Tumour type* | *Social class* % *I* | *II* | *III* | *IV* | *V* | *Not assigned* | *Total* |
|---|---|---|---|---|---|---|---|
| Adenocarcinoma $n=704$ | 1.14 | 10.65 | 23.29 | 8.81 | 2.70 | 53.41 | 100 |
| Squamous carcinoma $n=4599$ | 0.98 | 8.57 | 22.61 | 10.11 | 4.87 | 52.86 | 100 |
| Proportion of squamous carcinoma in each class | 0.849 | 0.840 | 0.864 | 0.882 | 0.922 | 0.866 | — |

**Table V** Association of suspected risk factors with adenocarcinoma compared with squamous carcinoma

| *Risk factor* | *Odds ratio* | *95% confidence limits* | *Adjusted for* |
|---|---|---|---|
| Cigarette use | 0.96 | (0.67–1.38) | Age, social class |
| Oestrogen/OC use | 1.25 | (0.73–2.11) | Age, social class, no. of preganancies |
| Diabetes | 1.61 | (0.85–3.08) | Age, smoking, social class |
| Hypertension | 2.23 | (1.12–4.43) | Age, smoking, social class |

**Figure 2** Survival from cervical carcinoma SE & SW Thames, 1968–81. Difference is significant (chi-square = 13.28, df = 1, $P<0.005$), adjusted for age and stage, chi-square = 6.97, $P=0.008$.

is not ideal but is appropriate when such controls are not available. Because of the rarity of cervical adenocarcinoma the patients had accrued over many years, and are a mixture of dead, newly diagnosed and prevalent cases. This in itself would raise methodological issues as to appropriate sources of controls. We chose squamous carcinomas accrued over the same period because, although recording of known risk factors for a given site is likely to be biased, this bias should be minimised if different histological types of the same cancer are compared. These may not even be distinguished until later in the registration process. Moreover, risk factors for squamous carcinoma are well-documented. Such a 'diseased' comparison group whose associations are well known may be used in two ways: the first is for direct comparison with the disease of interest and in this respect we have shown various similarities and differences. The second is to infer the association that would have been found if healthy controls had been chosen. For example, women in our two disease groups did not, overall, differ significantly with respect to social class although in women of known class there was a significant gradient in the proportion of adenocarcinomas with fewer cases being in lower social classes. We would therefore predict that in comparison with non-diseased controls, adenocarcinoma cases would show a weaker social class gradient than is found with cervical carcinoma in general, i.e., rates in social class V about twice rather than nearly four times those in class I (OPCS, 1981). Similar considerations apply to known cigarette smoking and use of oestrogens/oral contraceptives. Several reports exist of an association (not always significant) between cigarette smoking and invasive cervical carcinoma (Wright *et al.*, 1978; Wigle, 1980; Williams & Horm, 1977; Stellman, 1980; La Vecchia *et al.*, 1986). If we assume a relative risk of about 1.75 among smokers for all invasive cervical carcinoma – and hence for squamous carcinoma – we can predict the relative risk of adenocarcinoma among smokers would be about $0.96 \times 1.75 = 1.68$ (using our estimate of the association between smoking and tumour type, Table V). Similarly if we assume a relative risk of 1.11 in oral contraceptive users (WHO, 1985) we would predict the relative risk of adenocarcinoma in oestrogen/oral contraceptive users to be about $1.25 \times 1.11 = 1.39$. Because our data only referred to 'known use', misclassification is likely to have biased our results towards an underestimate. Nevertheless, such estimates of relative risk might be used to refine sample size calculations when planning further studies. Readily available, routinely collected information can thus be used under certain circumstances even without population controls, to infer aspects of the epidemiology of a cancer.

Only some 10% of our cases lacked specific histological typing and our results are broadly similar to those of others with respect to age, parity, marital status, social class and survival (Bergsjo, 1963; Korhonen, 1980; Menczer *et al.*, 1978; Milsom & Friberg, 1983; Tasker & Collins, 1974). Our finding, that hypertension but not diabetes was associated with adenocarcinoma rather than squamous carcinoma, is also consistent with that of Korhonen (1980). On the other hand while Milsom (1983) found that diabetes rather than hypertension was associated with adenocarcinoma, both conditions are associated with obesity and a hormonal basis for cervical adenocarcinoma remains a possibility as for endometrial carcinoma (MacMahon, 1974). Because these odds ratios are obtained from data of limited quality, some caution is needed in their interpretation. As described earlier they are conservative estimates. Hence while the 'non-significant' odds ratios may mask a real association, the true odds ratio for hypertension is probably greater than that shown in Table V.

With respect to extent of disease and age at presentation our results differ from those of Tasker and Collins (1974) and Bergsjo (1963). However, these studies may be faulted on the basis of small numbers and a possibly inappropriate comparison group. We were also unable to confirm similarities in the pattern of age-specific incidence rates for intraductal carcinoma of the breast and cervical adenocarcinoma noted by Henson (1977). The suggestion (Menczer *et al.*, 1978) that adenocarcinomas are more susceptible to time trends than squamous carcinomas was not supported by our data. The age adjusted decline in invasive adenocarcinoma registrations over 1968–81 was not significantly different from that in squamous carcinoma registrations and if anything was rather less, a finding consistent with greater difficulty in detection of adenocarcinoma by screening.

Our series also contains a relatively high proportion of adenocarcinomas compared with other large series of invasive cervical carcinoma, (WHO, 1985; Peters *et al.*, 1986). Many factors could be responsible including age structure, screening policy and different criteria of classification but we do not feel that the explanation lies in contamination of our results by misclassified endometrial carcinomas. Additional comparisons of cervical and uterine adenocarcinomas in STCR showed marked differences in age distribution, marital status, smoking habit and pill use indicating that the Cancer Registry records successfully distinguish these conditions.

For the future we suggest that studies should specify histological type wherever possible. The primary role of sexual behaviour and other possible risk factors such as smoking, oral contraceptives (Peters *et al.*, 1986) and papilloma viruses needs to be assessed as well as that of Herpes viruses (Menczer *et al.*, 1981; Wilkie *et al.*, 1980). The quality of histological diagnosis and its changes with time should also be studied in order to validate conclusions based on routine data.

We thank Mr Trevor Murrells of the Thames Cancer Registry for GLIM analyses.

### References

AITKEN-SWAN, J. & BAIRD, D. (1966). Cancer of the uterine cervix in Aberdeenshire. Epidemiological aspects. *Br. J. Cancer*, **20**, 624.

ALDERSON, M.R. (1985). Epidemiology of lung cancer from small cell carcinoma, *Clin. Oncol.*, **4**, 1.

ARMITAGE, P. (1971). *Statistical Methods in Medical Research*, Blackwell Scientific Publications, Oxford.

BAKER, R.J. & NELDER, J.A. (1978). *The GLIM System*. Release 3, Numerical Algorithms Group, Oxford.

BERGSJO, P. (1963). Adenocarcinoma cervicis uteri. A clinical study. *Acta Obstet. Gynecol. Scand.*, **42**, 85.

BOYD, J.T. & DOLL, R. (1964). A study of the aetiology of carcinoma of the cervix uteri. *Br. J. Cancer*, **18**, 419.

BUCKLEY, J., HARRIS, R., DOLL, R., VESSEY, M. & WILLIAMS, P. (1981). Case control study of the husbands of women with dysplasia or carcinoma of the cervix uteri. *Lancet*, **ii**, 1010.

CORREA, P., SASANO, N., STEMMERMANN, G.N. & HAENSZEL, W. (1973). Pathology of gastric carcinoma in Japanese populations: Comparisons between Miyagi Prefecture, Japan and Hawaii. *J. Natl Cancer Inst.*, **51**, 1449.

DOLL, R., BRADFORD HILL, A. & KREYBERG, L. (1957). The significance of cell type in relation to the aetiology of lung cancer. *Br. J. Cancer*, **11**, 43.

HARRIS, R., BRINTON, L., COWDELL, R., SKEGG, D., SMITH, P., VESSEY, M. & DOLL, R. (1980). Characteristics of women with dysplasia or carcinoma of the cervix uteri. *Lancet*, **ii**, 1010. **42**, 359.

HENSON, D. & TARONE, R. (1977). An epidemiological study of cancer of the cervix, vagina and vulva based on the Third National Cancer Survey in the United States. *Am. J. Obstet. Gynecol.*, **129**, 525.

KAPLAN, E.L. & MEIER, P. (1958). Non parametric estimation from incomplete observations. *J. Am. Stat. Assoc.*, **53**, 457.

KORHONEN, M.O. (1980). Epidemiological differences between adenocarcinoma and squamous carcinoma of the uterine cervix. *Gynecol. Oncol.*, **10**, 312.

LA VECCHIA, C., FRANCHESCHI, S., DE CARLI, A., FASOLI, M., GENTILE, A. & TOGNONI, G. (1986). Cigarette smoking and the risk of cervical neoplasia. *Am. J. Epidemiol.*, **123**, 22.

MACMAHON, B. (1974). Risk factors for endometrial cancer. *Gynecol. Oncol.*, **2**, 122.

MENCZER, J., MODAN, B., OELSNER, G., SHARON, Z., STEINTIZ, R. & SAMPSON, S. (1978). Adenocarcinoma of the uterine cervix in Jewish Women. A distinct epidemiological entity. *Cancer*, **41**, 2464.

MENCZER, J., YARON-SCHIFFER, O., LEVENTON-KRISS, S., MODAN, M. & MODAN, B. (1981). Herpes virus type 2 in adenocarcinoma of the uterine cervix: a possible association. *Cancer*, **48**, 1497.

MILSOM, I. & FRIBERG, L.G. (1983). Primary adenocarcinoma of the uterine cervix. A clinical study. *Cancer*, **52**, 942.

OPCS STUDIES ON MEDICAL AND POPULATION SUBJECTS NO. 43. (1981). *Cancer Statistics: incidence, survival and mortality in England and Wales*. HMSO, London.

PETERS, R., CHAO, A., MACK, T., THOMAS, D., BERNSTEIN, L. & HENDERSON, B. (1986). Increased frequency of adenocarcinoma of the uterine cervix in young women in Los Angeles county. *J. Natl Cancer Inst.*, **76**, 423.

PETO, R., PIKE, M.C., ARMITAGE, P. & 7 others. (1977). Design and analysis of randomised clinical trials requiring prolonged observation of each patient. II Analysis and examples. *Br. J. Cancer*, **35**, 1.

REEVES, W.C., BRINTON, L.A., BRENES, M.M. & 3 others. (1985). Case control study of cervical cancer in Herrera province, Republic of Panama. *Int. J. Cancer*, **36**, 55.

ROTKIN, I.D. & KING, R.W. (1962). Environmental variables related to cervical cancer. *Am. J. Obstet. Gynecol.*, **83**, 720.

SPRING, S. & GRUBER, J. (1985). Relationship of DNA viruses and cervical carcinoma: report of a workshop. *J. Natl Cancer Inst.*, **75**, 589.

STELLMAN, S.D., AUSTIN, H. & WYNDER E.L. (1980). Cervix cancer and cigarette smoking: a case control study. *Am. J. Epidemiol.*, **111**, 383.

TASKER, J.T. & COLLINS, J.A. (1974). Adenocarcinoma of the uterine cervix. *Am. J. Obstet. Gynecol.*, **118**, 344.

WHO ICD-O. (1976). *International Classification of Diseases for Oncology*. 1st edition. WHO, Geneva.

WHO. (1985). Collaborative study of neoplasia and steroid contraceptives. Invasive cervical cancer and combined oral contraceptives. *Br. Med. J.*, **290**, 961.

WIGLE, D.T., MAO, Y. & GRACE, M. (1980). Re: Smoking and cancer of the uterine cervix: Hypothesis. *Am. J. Epidemiol.*, **111**, 125.

WILKIE, N.M., EGLIN, R.P., SANDERS, P.G. & CLEMENTS, J.B. (1980). The association of herpes simplex virus with squamous carcinoma of the cervix and studies of the virus thymidine kinase gene. *Proc. Roy. Soc. London*, **210**, 411.

WILLIAMS, R.R. & HORM, J.W. (1977). Association of cancer sites with tobacco and alcohol consumption and socioeconomic status of patients: Interview study for the Third National Cancer Survey. *J. Natl Cancer Inst.*, **58**, 525.

WRIGHT, N.H., VESSEY, M.P., KENWARD, B., MCPHERSON, K. & DOLL, R. (1978). Neoplasia and dysplasia of the cervix uteri and contraception: a possible protective effect of the diaphragm. *Br. J. Cancer*, **38**, 273.